# Are trauma patients better off in a trauma ICU?

**DOI:** 10.4103/0974-2700.43183

**Published:** 2008

**Authors:** Therese M Duane, Ivatury R Rao, Michael B Aboutanos, Luke G Wolfe, Ajai K Malhotra

**Affiliations:** Department of Surgery, Division of Trauma, Critical Care and Emergency General Surgery, Virginia Commonwealth University Medical Center, VA 23298

**Keywords:** ICU, intensivist, outcomes, trauma

## Abstract

There is very little data on the value of specialized intensive care unit (ICU) care in the literature. To determine if specialize ICU care for the trauma patient improved outcomes in this patient population. Level I Trauma Center Compared outcomes of trauma patients treated in a surgical trauma ICU (STICU) to those treated in non- trauma ICUs (non-STICU). Retrospective review of trauma registry data. Statistical Analysis: Wilcoxon Rank Test, Fischer's Exact test, logistic regression. There were 1146 STICU patients compared to 1475 non-STICU. In all ISS groups there were more penetrating trauma patients in the STICU (32.54% STICU vs. 18.15% non-STICU, *P*<0.0001 (ISS< 15)), (21.03% STICU vs. 12.98% non-STICU, *P*=0.0074 (ISS between 15-25)), and (19.42% STICU vs. 11.35% non-STICU, *P*=0.0026 (ISS> 25)). All groups had similar lengths of stay. The blunt trauma patients were sicker in the STICU (20.8 ISS ± 12.2 STICU vs. 19.7 ISS ± 11.9 non-STICU, *P*=0.03) yet had similar outcomes to the non-STICU group. Logistic regression identified penetrating trauma and not ICU location as a predictor of mortality. Sicker STICU patients do as well as less injured non-STICU patients. Severely injured patients should be preferentially treated in a STICU where they are better equipped to care for the complex multi-trauma patient. All patients, regardless of location, do well when their management is guided by a surgical critical care team.

## INTRODUCTION

Modern trauma care has become highly specialized, especially for the critically ill patient with multi-system injuries. These patients require specialized care that can only be provided by specially trained nurses and support staff. In most busy trauma centers the critically ill patients exceed the available surgical trauma intensive care unit (STICU) beds, forcing appropriate “shuffle” of patients to the STICU vis-a-vis the other ICUs.

The only literature available on this subject comes from the neurosurgical intensive care. Mirski *et al.*[1] retrospectively compared intracerebral hemorrhage patients and found those treated in a specialized neuroscience ICU had lower mortality, length of stay and cost than those treated in a general ICU. We analyzed our results of a triage algorithm that sorted critically ill trauma patients into those that absolutely require STICU expertise and patients of lesser needs and acuity to non STICU units.

## MATERIALS AND METHODS

Logistic considerations: Our Level I Trauma center (state certified and ACS-COT verified) admits approximately 3500 patients a year. All trauma patients who require ICU admission, were triaged by the trauma attending to be admitted to STICU and non-STICU ICUs (in our institution these are; neuroscience, medical/respiratory, cardiac, cardio-thoracic ICUs). The criteria that mandate a STICU admission, as opposed to a non-STICU placement are listed in [Table 1]. Isolated head injury patients are preferentially admitted to the Neuroscience ICU as are patients with severe head injury requiring invasive cerebral monitoring and open abdomens.

**Table 1:** Indications for STICU admission

Hemodynamic instability requiring vasoactive drips
Fluid resuscitation needing invasive monitoring
Severe hypoxia necessitating complex ventilatory management
High risk patients defined by 2 or more of the following:
multisystem trauma
complex pelvic fractures
elderly with pre-existing coronary, pulmonary or peripheral vascular disease
Patients status post damage control surgery with open abdomens or chests
Actively bleeding and/or coagulopathy requiring aggressive blood/fluid replacement or correction of hypothermia
Significant penetrating trauma to chest and/or torso

STICU: Surgical Trauma Intensive Care Unit

Regardless of the ICU location, all these trauma patients are managed by a multidisciplinary team that includes a surgical intensivist, surgical ICU fellow, rotating second year general surgery residents, surgical subspecialty interns, emergency department residents, a doctor of pharmacology, nutritionist, and an ICU nurse practitioner. Patients have similar protocols and management guidelines for sedation, glycemic control, ventilator weaning and central lines.

For this study we retropsectively analyzed all trauma patients 16 years or older who were admitted directly to the ICU from the emergency department, immediately after initial operation or at any time during their hospital course. We excluded pediatric ICU admissions, burn ICU admissions and patients with isolated severe closed head injury treated in the neuroscience ICU. Polytrauma patients who were originally placed in the neuroscience ICU and subsequently transferred to the STICU were included. The group of patients that originally began their care in a non-STICU and subsequently transferred into the STICU were considered as an intention to treat group and included in the non-STICU group for purposes of statistical analysis.

We evaluated the groups for initial demographics and outcome measures which included infectious complications, ventilator days, ICU days and mortality rates. The infectious complications analyzed were: urinary tract infection (UTI), bacteremia, pneumonia, and wound infection. UTI was defined as a positive urine culture of greater than 100,000 organisms per high power field (hpf). Bacteremia required two positive blood cultures for diagnosis, and pneumonia was diagnosed by a combination of findings which included new infiltrate on chest radiograph, increasing white blood cell count, fever, purulent sputum and in all intubated patients, bronchoscopy and bronchoalvealar lavage demonstrating 10^5^ colony forming units (CFU). Wound infections were diagnosed by direct inspection and culture confirmation when available.

Continuous variables were evaluated using the Wilcoxon Rank Test and the nominal variables were evaluated using the Fischer's Exact test. Further logistic regression was performed to determine the effect that mechanism of injury had on outcome. This study was approved by the Virginia Commonwealth University Institutional Review Board.

## RESULTS

During the study period 6926 patients had trauma team activation at our level 1 trauma center. 1146 patients were admitted to the STICU during their hospital stay and 1475 patients were admitted to a non-STICU. These included 290 patients who were admitted to a non-STICU and subsequently transferred to the STICU. There were 274 patients who were admitted directly to the STICU after emergent or urgent operative intervention shortly after arrival at the trauma center versus 175 admitted to non-STICU's. Open abdomens were followed beginning in 2001 and since that time seventy-seven patients were treated in the STICU with open abdomens versus only 36 in outlying units. Overall 51% of STICU patients versus 46.5% of non-STICU patients had an operation (*P*=0.04) while 6.5% of STICU and 2.4% of non-STICU patients (*P*<0.0001) had an open abdomen. 29 of the 36 in the non-STICU group were immediate ICU admissions and the majority of these (18/29) had concomitant severe head trauma requiring invasive cerebral monitoring.

The two groups had similar co-morbidities at the time of injury. ISS, calculated retrospectively, was higher in the STICU group (19.7 ± 12.2 STICU vs. 18.1 ± 12.2 non-STICU, *P*=0.0007), confirming the triage decision of sicker patients to the STICU. There were a total of 2090 patients with blunt trauma, 1244 in the non-STICU group and 846 in the STICU group. They were of similar age (42.3 years ± 18.6 STICU vs. 43.6 years ± 20.3 non-STICU, *P*=0.41), but the STICU group was more severely injured with a higher ISS (20.8 ± 12.2 STICU vs. 19.7 ± 11.9 non-STICU, *P*=0.03) and new ISS (28.2 ± 16.9 STICU vs. 25.8 ± 16.2 non-STICU, *P*=0.0023). Overall 51% of STICU patients versus 46.5% of non-STICU patients had an operation (*P* =0.04). Other than more infectious complications in the STICU group (15.7% (133/846) STICU vs. 10.9% (136/1244) non-STICU), the remaining outcome measures were the same as shown in [Table 2].

**Table 2:** Outcome measures for blunt trauma patients

	STICU	Non-STICU	*P*value
% Bacteremia	7.9(67/846)	5.9(73/1244)	0.07
% Pneumonia	5.9(50/846)	5.1(63/1244)	0.43
% UTI	4.49(38/846)	3.94(49/1244)	0.58
% Wound	4.02(34/846)	1.77(22/1244)	0.0022
Ventilator length of stay (days)	2.2 ± 6.8	2.6 ± 7.1	0.58
ICU length of stay (days)	6.2 ± 11.2	5.5 ± 8.8	0.37
Hospital length of stay (days)	14.4 ± 21.2	11.5 ± 13.5	0.71
% mortality	9.2(78/846)	8.2(102/1244)	0.43

STICU: Surgical Trauma Intensive Care Unit

There were 504 documented penetrating trauma patients with 290 in the STICU and 214 in the non-STICU. In all ISS groups there were more penetrating trauma patients in the STICU (32.54% STICU vs. 18.15% non-STICU, *P*<0.0001 (ISS< 15)), (21.03% STICU vs. 12.98% non-STICU, *P*=0.0074 (ISS between 15-25)), and (19.42% STICU vs. 11.35% non-STICU, *P*=0.0026 (ISS> 25)). 58.9% of penetrating trauma patients had operative treatment, and over 11% had open abdomens. Other than a higher mortality rate in the STICU (11.03% (32/290) STICU vs. 5.14% (11/214) non-STICU, *P* =0.02), the rest of the outcome measures were similar as shown in [Table 3].

**Table 3:** Outcome measures for penetrating trauma patients

	STICU	Non-STICU	*P* value
% infections	19.66(57/290)	18.69(40/214)	0.82
% Bacteremia	10.0(29/290)	9.8(21/214)	1.0
% Pneumonia	5.17(15/290)	5.6(12/214)	0.84
% Urinary tract infection	3.45(10/290)	4.21(9/214)	0.81
% Wound	8.28(24/290)	7.01(15/214)	0.62
Ventilator days	2.0 ± 5.1	2.4 ± 5.9	0.68
ICU days	5.9 ± 12.3	6.3 ± 11.3	0.84
Hospital length of stay (days)	13.3 ± 17.7	14.8 ± 20.6	0.78

STICU: Surgical Trauma Intensive Care Unit, ICU: Intensive Care Unit

Logistic regression demonstrated that ISS (OR 1.062, CI 1.053-1.070), penetrating trauma (OR 2.6, CI 2.072-3.300), age (OR 1.019, CI 1.014-1.024) and ICU location (OR 1.705, CI 1.424-2.041) were all predictors of complications (r^2^=0.1770, *P*< 0.0001). ISS, age and complications were also predictive of mortality with a p value of <0.0001. Penetrating trauma remained a predictor of mortality (*P* = 0.0002 (r^2^ =0.34)) [Table 4] but ICU location did not.

**Table 4:** Logistic regression on outcome

	Odds ratio	Confidence interval
Injury severity score	1.080	1.066-1.095
Age	1.038	1.029-1.047
Complications	5.195	3.659-7.376
Penetrating trauma	2.228	1.445-3.437

## DISCUSSION

The role and the type of ICU has received very little attention in the literature when analyzing outcomes from critical injuries. This oversight is a serious problem in an era of increasing severity and frequency of multi-system injuries and the almost daily occurrence of “spill-over” of these patients into non-specialized units, where inadequately trained health care staff are called upon to provide specialized care. Seasoned nurses’ ability to aggressively manage patients and function independently is a requisite for optimal trauma care. The average experience for a nurse in our STICU is 12 years. Just under half of the nurses (48%) have worked in this unit between 10 and 30 years. Complex, high acuity nursing interventions such as volume replacement, correction of coagulopathy and hypothermia, invasive monitoring and the management of “damage-control” situations require knowledge and skills that are not quantifiable. These skills are obtained on a daily basis in Trauma ICU s where there is an abundance of “hands-on” learning opportunities. The development of such skills is essential for optimal results in life-threatening blunt and penetrating trauma. An equivalent care is hard to obtain even from staff that is experienced and outstanding in their non-surgical fields (e.g. cardiac units)

For this reason, our triage scheme of severely injured patients, directs the most complex patients to the STICU in an effort to optimize their ICU treatment. The example has been set in the neuroscience population. Mirski *et al.*[1] showed improvements across the board when patients with intracerebral hemorrhage were treated in a specialized neuroscience unit. These findings were replicated by Diringer and Edwards.[2] In their study forty-two ICU's were included in a prospective study. They, too, found that patients with intracerebral hemorrhage had a lower mortality when treated in a NSICU supporting the argument for a specialized ICU for these patients. It is surprising that such studies have not been performed in a patient population with major trauma.

Our practice of preferentially selecting the most severely injured patients and those with life threatening penetrating trauma to the STICU appears to be providing optimal results. Even though the injury severity of the STICU and non-STICU groups appear comparable, it is likely that the patients who go to the STICU are actually sicker which cannot be measured with a purely anatomic scoring system of injury severity. Also of note, in all the injury severity groups a greater number of penetrating trauma subjects were in the STICU and these patients tend to have higher acuity requiring more advanced therapeutic interventions, damage-control procedures etc.[3] Our data confirmed that patients in the STICU and penetrating trauma patients had more operations and more open abdomens. Our regression analysis for mortality suggests that penetrating trauma is a predictor of mortality which also supports the above supposition.

It is also of interest that in the blunt trauma subset of patients the STICU had statistically sicker patients yet their outcomes were the same as the non-STICU group. The STICU group also preferentially had more open abdomen patients, which may explain the higher infection rate observed in this group. A recent paper by Levy *et al.*[4] had a similar conundrum demonstrating that significantly sicker patients were treated by intensivists. Even with aggressive propensity matching the intensivist group had higher mortality rates. Other non-quantifiable pertinent factors included: intensivist-based care was overwhelmingly provided at academic centers where patients with more co-morbidities tend to seek treatment, and more transfers from outside institutions came to ICU's with intensivist coverage. The majority of these patients are too high acuity for smaller institutions. Therefore, similar to the Levy paper, our patients were probably disproportionately sicker yet this could not be demonstrated statistically somewhat skewing the results.

We believe that the intensivist model we use may have contributed to our results. Regardless of physical location, all of our patients were treated by an ICU team lead by a surgeon with added qualifications in surgical critical care and carried out by experienced nursing staff. Unlike the Levy paper, most studies have found that the intensivist model improves outcomes independent of location, especially in patients who do not require the most advanced nursing trauma care. This approach has been borne out by recent emphasis on an intensivist model of ICU care. Young and Birkmeyer[5] looked at the potential reduction in mortality rates when intensivists were constantly available. They based their calculations on the Leapfrog Group's recommendation. Review of nine studies demonstrated a relative reduction in mortality ranging from 15% to 60% which approximated to 53,850 lives saved. Such intensivist models shows benefit across specialties. Suarez *et al.*[6] focused on a neuroscience ICU population. They found that the presence of a neurocritical care team was an independent predictor of decreased in-hospital mortality and hospital length of stay. Russell *et al.*[7] evaluated outcomes in a neuroscience ICU after acute care nurse practitioners were added to the team. The patients managed by the nurse practitioners had shorter lengths of stay and lower urinary tract infections with cost savings over two million dollars. Nathens *et al.*[8] used data from a large multicenter study to evaluate the relationship between an open versus an intensivist model for trauma ICU care. The intensivist model included both closed units where management was led by an intensivist and units where the patients were co-managed with an intensivist. They found a large reduction in in-hospital mortality following trauma particularly in the elderly population. In a study that pooled data from a broad range of ICU's, Provonost *et al.*[9] demonstrated that ICU's where all care was directed by the intensivist had reduced ICU and hospital mortality as well as ICU and hospital length of stay. Others studies have demonstrated the value of the intensivist even when he/she is not physically present. Rosenfeld *et al.*[10] performed an observational trial in which comparisons were made from a time when intensivists were available as needed to a time when they were continuously present via remote monitoring including video conferencing and computer-based data transmission. They showed a decrease in complications, ICU length of stay, ICU costs, and mortality.

Our study supports the concept of specialized ICU care, appropriately triaged between Trauma and non-Trauma ICUs. The more severely injured blunt trauma patients, life-threatening penetrating trauma victims, “damage-control” situations, multi-system trauma with the potential for multi-organ failure and patients at risk for massive transfusion, abdominal compartment syndrome, acute respiratory failure, extremity compartment syndromes should be preferentially placed in the Trauma ICUs. It is this select group of severely injured patients that require the years of experience in complex trauma care that only a Surgery/ Trauma ICU can provide. ICU location may not be as important for the less severely injured patients, as long as their management is guided by an ICU team consisting of a surgical intensivist and dedicated, experienced nursing personnel. Triage of the most severely injured patients to a dedicated STICU is crucial for ensuring optimum survival.
